# Active Ageing in Italy: An Evidence-Based Model to Provide Recommendations for Policy Making and Policy Implementation

**DOI:** 10.3390/ijerph19052746

**Published:** 2022-02-26

**Authors:** Davide Lucantoni, Andrea Principi, Marco Socci, Marina Zannella, Francesco Barbabella

**Affiliations:** Centre for Socio-Economic Research on Ageing, National Institute of Health and Science on Ageing (IRCCS INRCA), 60124 Ancona, Italy; d.lucantoni@inrca.it (D.L.); a.principi@inrca.it (A.P.); m.zannella@inrca.it (M.Z.); f.barbabella@inrca.it (F.B.)

**Keywords:** policy recommendations, European national strategies, active ageing, evidence-based policy making

## Abstract

In ageing societies, active ageing (AA) has been recognized as a useful conceptual tool due to its holistic approach to social issues and recognised benefits from it at multiple levels (micro, meso and macro) for addressing demographic challenges. However, one of the main problems identified in relation to AA, is to turn into practice, at the policy making level, the many positive aspects that it promises at the conceptual level, as is demonstrated by the available evidence based on experiences carried out in some European countries. As an advancement in this field, through an original research experience carried out in Italy between 2019 and 2021, this study for the first time provides a model for producing recommendations for policy making and policy implementation in the field of AA, by managing the main problematic aspects related to the operationalization, at the policy making level, of the AA concept, with the potential for replication in other countries. The main challenges were identified, as well as the way to deal with them through a model, for a proper operationalization of the AA concept, based, among other aspects, on a solid international framework concerning this matter, on a mainstreaming ageing approach (at the public policy level) and on a wide stakeholder participation through co-decisional tools. A multi-level (national-regional-local) perspective was adopted to consider cultural and geographical diversity, among other challenges.

## 1. Introduction

In the past few decades, the trend of population ageing has progressively come to the attention of both national and international institutions, raising new challenges in social, political, economic and cultural spheres of global communities. By 2050, older people will represent 22% of the world population, and in the next five years the number of people aged 65 or over will be higher than that of children having less than five years [1]. The increase in longevity is a positive achievement of our times, with several implications (e.g., labour market, social cohesion, health, welfare systems and public spending) resulting in the emergence of new social and policy needs. This should be addressed through an urgent and adequate commitment at the policy, socio-economic, health and research level, in order to maintain and increase the sustainability of the ageing process [2]. In this framework, the active ageing (AA) concept represents a useful tool, due to its holistic approach to social issues and the recognised benefits from it at all levels, in order to address the challenges posed by population ageing.

AA is defined as “the process of optimizing opportunities for health, participation and security in order to enhance quality of life as people age” [3] (p. 12). At the micro level, several international studies have shown the positive link existing between AA and physical/psychological health, resulting from the formal or informal activation of the older person in social or personal areas [4,5]. At the macro level, the promotion of AA allows fostering of the sustainability of the welfare system in terms of reduction of public spending for assistance and social-health care interventions, enhancement of the productive contribution deriving from the activities undertaken, as well as balancing the social security system [6]. Because of the benefits from it at various levels, AA can be considered as a win-win solution [7].

Through the AA paradigm, older people are considered as resources for the community and the social system as a whole [8], by empowering them to engage in activities coherent with their motivations and aspirations, with the aim to overcome the unfruitful perspective and approach of considering older people as just care receivers or passive citizens [9]. In this perspective the AA model has been built by the World Health Organization (WHO) encompassing determinants which are addressed, at the global level, through—among other provisions—the adoption by the WHO 69th World Health Assembly of the Global Strategy and Action Plan on Ageing and Health in 2016 [10]. The latter aimed to develop a renewed action plan aligned with the Sustainable Development Goals (SDGs) of the Agenda 2030 for Sustainable Development [11]. In consideration of this, AA is widely regarded as a fundamental policy concept in Europe [12], where it is promoted within the Regional Implementation Strategy (RIS) of the Madrid International Plan of Action on Ageing (MIPAA) [3], along with multiple documents and initiatives such as the European Year of Active Ageing and Solidarity between Generations in 2012 [13], the European Innovation Partnership for Active and Healthy Ageing (EIPAHA) and the Green Paper on Ageing [14].

## 2. Policy Making in the Field of Active Ageing: Main Challenges and Conceptual Framework

Despite its several positive aspects, a serious difficulty concerning AA is to move from theory to practice, since the application of the concept at the policy level up to now has often been considered misleading [15]. The literature highlights the main challenges that should be addressed to avoid misinterpretations when developing and implementing policies if this field [16,17,18,19,20]:

C1. In order to deal with policy making in this field and being sure not to neglect the many interconnected aspects, national applications of the AA concept should be in line with a proper and strong AA comprehensive international framework to ensure its homogeneity and success at the wider international level. Policy makers should concentrate their attention and efforts on the heterogeneity and diversity of experiences of later life, relying on conceptual frameworks in which this diversity is modelled, as the outcome of a lifetime’s interaction between a set of intrinsic capacities of individuals and the social environment in which they live [18]. In this perspective, AA frameworks should advocate a holistic approach, arguing that multiple aspects of older adults’ activities, specifically participation, health, security, psychological well-being, lifestyle and financial resources intertwine to determine the quality of the ageing experience, and that each of these aspects are essential in achieving and maintaining well-being in later life [21].
C2. With the aim to avoid sectorial approaches of policy making in the area of AA, usually confined to social services and health departments, the concept of mainstreaming ageing across all policy domains should be applied through a systematic integration of AA issues across all relevant policy fields [17]. Mainstreaming ageing has become a priority on policy agendas throughout Europe, but it is still not fully understood and implemented [22]. Thus, undeniable national achievements in various ageing-specific sectors co-exist with significantly less progress in mainstreaming ageing into wider policy discourse and development strategies. For instance, there is still a substantial gap between the comprehensive philosophy of the UN Madrid International Plan of Action on Ageing (MIPAA) and the related commitments for its implementation [23] and the sectoral translation of the MIPAA commitments into national programs [24]. A real involvement of public authorities at various levels is needed. Different stakeholders across different policy areas are compelled to work together on designing effective and comprehensive strategies for active ageing [25]. When social policy goals aimed at older persons are incorporated into overall national development plans, this integration may have a positive long-term effect on the well-being of the older population [24].
C3. It has also been observed, as a criticism, that AA is politically pursued mainly through a top-down approach. See for example: https://statswiki.unece.org/display/AAI (accessed on 10 January 2022), while AA should not be a top-down imposition, but rather opportunities should be provided for citizens to take action from the bottom-up, through consultative and/or co-decisional participative tools. This is highly important in the perspective of producing socially innovative solutions in order to solve social problems [26,27]. The effectiveness of the stakeholder involvement depends on its capacity to address local inputs through a coordinated and multilevel approach among public institutions and actors from civil society and the Third Sector [28]. According to this, horizontal networks of public, private, and non-profit organizations provide new structures of governance, as opposed to hierarchical organizational decision making [29]. In fact, stakeholder and user involvement is often limited to the collection of citizen’s opinions about a specific issue, and frequently results in low-profile deliberations, while co-decisional approaches set the conditions for a deliberative dialogue between governors and governed [20]. This would allow to better consider motivations, preferences and constraints of older people by also ensuring sustainability of policy making in this field. The latter is often and seriously jeopardized when participative tools are implemented without providing or enforcing coordination and informative systems among national, regional and local levels, resulting in a lack of continuous involvement of the non-governmental organizations of seniors in the consultation process [30].
C4. The widespread exercise of comparing different contexts in terms of AA has been largely criticized [31], while scientists have underlined the strong need of respecting national and cultural diversity in implementing AA policies [16], since older people’s values and aims related to AA dimensions may vary considerably depending on the studied context, culture, and traditions [32], which can refer either to differences between countries or within the country. Therefore, the concept should be applied by respecting cultural diversity concerning activity patterns and norms. This is intended not only across countries, but also at the sub-national level (e.g., across country-regions) [33,34]. In fact, within countries, there are also large variations between ethnic/social groups in their preferences for different forms of activity [16]. In this regard, policy making on AA should pay attention to the local situation, and expect differences also in health, participation and security [35]. Respecting national and cultural diversity is of paramount importance to achieve the full potential of AA, without condoning practices that transgress national and international equality and human rights objectives and laws [36].
C5. Another issue concerns the limited efforts to activate older people who face more barriers in accessing AA, is those with less resources. AA initiatives should consider inequalities by providing opportunities for all people, including those with less available resources [32]. To achieve this goal, it is important to explicitly address gender, social class and other inequalities among older cohorts [37]. The lack of personal (i.e., social, human, cultural) resources represents a barrier to AA: as people grow older, disparities in terms of both wealth and other socio-economic resources are accentuated, with inequalities grounded in gender, class, ethnicity, sexual orientation, health and other aspects [38]. Actions that dismantle discrimination, and level up socio-economic conditions, will likely uplift the trajectory of healthy and active ageing for all people. In particular, in order to reduce accumulated inequalities, the following areas should be addressed by policy interventions: structures, norms and processes that shape and differentially affect a person’s likelihood to age well across the life course; integration across health and social care systems to mitigate vulnerabilities, and strengthen social capacities and abilities; measuring and assessing the impact of actions to better describe and identify inequities, and evaluate what initiatives work in different contexts [39,40].
C6. A further challenge is moving from an age-segregation to an age-integration perspective, through policy making in this field. This means taking into account the life-course perspective, through intergenerational exchange [41], promoting an intergenerational approach as a promising measure to support development and use of potentials of generativity in older people, in the interest of the old, the young, and the whole society [19]. The application of the concept should not generate competition between generations, but rather, since ageing is a process which people experience independently by their age, the concept should be applied in a life-course perspective. An intergenerational approach to AA does not consist of linking it only to intergenerational solidarity, but also to intergenerational relationships [42]. In fact, intergenerational solidarity often recalls the idea of mutual assistance, while intergenerational relationships are more referred to the structural interdependence of all individuals, as part of the society. According to this, the maintenance of an intergenerational perspective is considered as an important feature of a modern approach to AA in order to develop “fairness between generations as well as the opportunity to develop activities that span the generations” [43] (p. 125). It is through the encounters between ageing as a life-course process and generations as social relationships that intergenerational ageing becomes effective [42].
C7. One of the main criticisms is that policy making in this field addresses mainly employment-related issues, neglecting other areas of social activation. Thus, there is the need to avoid a purely economic/productive interpretation of AA in terms of only improving the labour market participation of older people. Indeed, it is also important promoting other meaningful activities (besides paid work) that can contribute to the well-being of older individuals and societies at large (e.g., volunteering). Although a comprehensive approach on AA was originally developed by the WHO and the United Nation (UN), a productivist approach focused on employment seems to be still dominant [16]. The productivist model tends to promote rational policy behaviour based on “objective and precise value-neutral calculations of the cost-benefits of policy changes” [44] (p. 236), which has the potential to reduce older persons to homos economicus and to means for utilitarian ends [45]. Such utilitarian treatment of the productive dimension of AA tends to render productive accomplishment equivalent to personal worth [46], obstructing other pathways for personal development than the work-based pathway [47]. In this regard, as noted by the WHO, a holistic approach to AA should pursue continued “participation in social, economic, cultural, spiritual and civic affairs, not just the ability to be physically active or participate in the workforce” [3] (p. 12).

The main challenges for policy making in the field of active ageing as identified above through the literature, are summarized in Table 1, where the actions needed to face them are reported.

**Figure 1:**
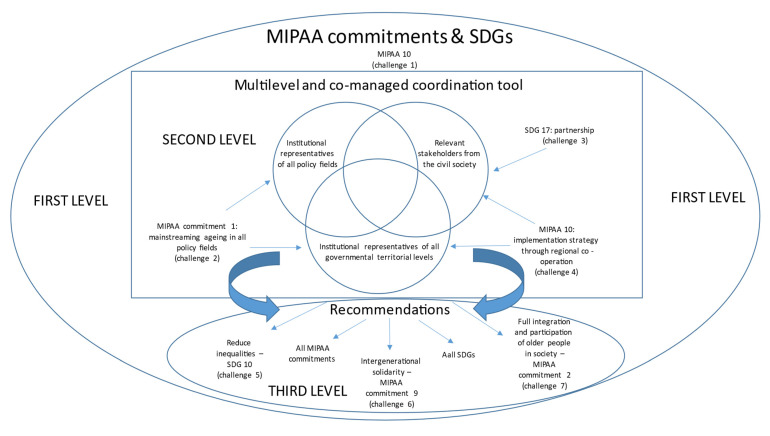
Conceptual framework.

These challenges concern three main levels, which converge in the conceptual framework shown in Figure 1.

The first level concerns C1, and it is about a solid international policy framework under which the recommendations should be developed. To be sure that all the main aspects are taken into account, the international framework adopted is represented by the MIPAA and its 10 commitments (Table 2). Since the latter has been largely connected to nine out of the 17 Sustainable Development Goals of the UN 2030 Agenda for Sustainable Development [48], these SDGs are also included in the international framework.

The second level concerns C2, C3 and C4, and has to do with organising the governance by starting to apply the commitments and the SDGs; a place where the many stakeholders interact to agree on the recommendations. This coordination tool should include institutional representatives of all policy fields in a mainstreaming ageing perspective (C2, addressed through MIPAA commitment 1); relevant representatives of the civil society to guarantee participation in co-decisions and be sure to prevent a top-down approach (C3, this issue considered by MIPAA 10 and SDG 17), and to consider regional diversity, these representatives should not only concern the national level but also the regional level (C4, in application of MIPAA 1 and MIPAA 10).

The third level concerns C5, C6 and C7, i.e., the outcome of the governance tool, that is the recommendations, to deliver by considering all the ten MIPAA commitments and the nine SDGs considered. Among them, some address specifically the other three main challenges identified: SDG 10 underlines the importance to guarantee active ageing opportunities considering inequalities (C5); MIPAA 9 on intergenerational relationship and solidarity addresses the important issue of the life-course perspective (C6) and MIPAA 2 warns about considering all possible societal fields of activation (C7).

## 3. Active Ageing from Theory to Practice: Examples of National Active Ageing Strategies

The main aim of this section is to understand whether the active ageing concept has been operationalized in Europe through national strategies or similar initiatives, and, if yes, to analyse the extent to what these experiences are consistent with the framework (or elements of the framework) described above. A review was conducted which highlighted some cases of European countries (i.e., Latvia, Malta, Bulgaria, Slovenia, Ireland, Spain and Czech Republic), which in recent years have tried to operationalize, politically, the concept of AA through national strategies, (Table 3).

### 3.1. Describing the Strategies

The national strategies presented were developed along the last decade. Most of them ended few years ago (i.e. Latvia, Czech Republic, Ireland, Malta, Spain), and in-formation concerning whether they continued through other following policies, are not always clear. 

About the Czech Republic’s strategy, it seems that the lack of cooperation between different administrative bodies, financial restrictions and the negative perception of the term ‘senior’ adopted, have limited the possibilities for implementing effectively the strategy. In this country a small number of measures promoting AA seem to have emerged accidentally or as a response to short-term needs [56].

In Ireland, after the 2013 strategy, a Healthy and Positive Ageing Initiative (HaPAI) (2014–2017) was established to implement the research objectives, during which the “Healthy and Positive Ageing for All” research strategy (2015–2019) was launched with the aim of supporting and promoting research in the area of ageing.

According to Sidorenko (2021) [57], the Latvian 2014–2016 strategy was not adopted by the legislative or governmental authorities, and the formulated elements of the AA policy were included in national policy documents of a wider profile, as in the national development strategies.

About the Spanish “Framework of action for older persons” a specific implementation approach has not been materialized from it yet [58].

The Slovenian and the Bulgarian strategies are still ongoing. According to the available information, the ageing issues have not been comprehensively addressed at the national level in Slovenia so far; therefore, great efforts will be needed to prepare concrete action plans and to effectively connect a large number of stakeholders [59]. With regard to the Bulgarian strategy, its implementation and sustainability are characterized by limited financial resources, low awareness of the problems of the older population in local communities, lack of appropriate infrastructure and poor intersectoral cooperation [60].

The Maltese national strategy has received, in general, positive reviews from the academic literature [61], in particular for its success in enabling higher rates of employment, participation in society and independent living amongst persons aged 60 and over. However, it has been pointed out that, at the same time, these areas were addressed through too many fragmented actions [62]. In order to create a new national political strategy for active ageing 2021–2027, a series of public consultation webinars on these topics were set up at the end of 2020.

Out of all the national AA strategies concerned, Maltese and Latvian ones have developed a set of policy recommendations, while in the Slovenian strategy guidelines were developed, and in the Spanish one a set of proposals were developed. Both guidelines and proposals were created by governmental institutions, with the difference that the Slovenian guidelines are considered as a binding tool for the public policy maker: “the individual ministries will draw up action plans and propose specific solutions to implement the guidelines. These action plans must be consistent in order to facilitate accomplishment of the objectives” [55]. The Spanish proposals, on the other hand, are considered as guiding principles open to future development or additions [56]. For that reason, both the Spanish proposals and the Slovenian guidelines are considered consistent with the definition of policy recommendations provided by Majeski and Sylvan (1999) [63].

Since most of the identified strategies have encountered some kind of problem in the operationalization of the AA concept at the policy level, we aim to explore the extent to what these strategies considered the main challenges mentioned above, which may have influenced the successful implementation of the strategies.

### 3.2. Analysing the Strategies

In the analysed strategies, the main challenges appear to be for the most part partially covered or uncovered (Table 4).

The use of an international framework to lay the foundations of the national AA strategy (C1) was considered by all the national strategies analyzed. Most of them (e.g., the Bulgarian, Latvian, Maltese, Slovenian and Spanish strategies) relied on the Active Ageing Index (AAI) framework. The Active Ageing Index (AAI) is an instrument to produce an evidence based on AA by measuring unused potentials of older people and by monitoring overall progress with respect to AA [64]. It is based on 22 outcome and context indicators, taking into account the particularities and characteristics of different geo-governmental and socio-economic contexts, which focuses on four domains (1) employment; (2) participation in society; (3) independent, health and secure living; (4) capacity and enabling environment for AA. The AAI was developed in 2012 by an initiative of the United Nations Economic Commission for Europe (UNECE) and the European Commission (EC). The scientific community recognized some weaknesses concerning this tool, among which was not covering properly the multidimensionality of AA and of being scarcely theory-driven [65,66]. The Czech and the Irish strategies were based on the MIPAA/RIS framework. The MIPAA framework is a comprehensive list of 10 commitments for United Nations Member States and focuses on three priority directions: older persons and development; advancing health and well-being into old age and ensuring enabling and supportive environments. In September 2002, the UNECE Member States adopted a Regional Implementing Strategy (RIS) in order to focus on the specificities of the demographic and economic situation in the region, which are, in many ways, different to other regions of the world. The UNECE’s MIPAA framework [3], which guided the strategies in Ireland and in the Czech Republic, represents a more appropriate political framework for responding to the opportunities and challenges of population ageing in a global and comprehensive way.

Mainstreaming ageing (C2) is considered in the Irish strategy, in which it is clearly stated that its full implementation should be considered as a first step for the mainstreaming of ageing in all policy fields in coherence with the MIPAA/RIS framework of reference. The Bulgarian strategy also includes the mainstreaming of AA as a general principle, but its effectiveness is limited to the four policy fields identified by the AAI framework of reference. In the other examples of our study, mainstreaming AA in all policy fields was not considered.

Stakeholder involvement and participation (C3) was considered, but to a limited extent, in all the strategies, whose development was guided by a consultation process. As an exception, in Slovenia no stakeholder network was involved in the development and coordination phases, which was carried out by governmental bodies. Nevertheless (even if so far there is no evidence about its implementation), in this strategy the involvement of a stakeholder network is planned in the future. In the Latvian case, apart from the initial development of the strategy, in the subsequent implementation phases, stakeholder participation (through co-decisional or consultative tools) was not particularly considered. These phases were mostly intended to be carried out only by governmental bodies (e.g., ministries, regions and municipalities). This may hinder the sustainability of the strategy. Instead, the Bulgarian strategy mentions (through an interinstitutional working group) the presence of coordination tools and co-decisional approaches during the implementation phase; however, the sustainability was jeopardized by for the reasons set out above (i.e., limited financial resources, low awareness of the problems experienced by the older population in local communities, and lack of appropriate infrastructure and poor intersectoral cooperation). In essence, despite in many strategies (such as the Spanish, Irish, Bulgarian, Czech and Maltese ones) sustainability being stimulated since their development phases through the stakeholder involvement in a consultation process, in none of them, except for the Bulgarian one, were identified long-term participative tools and informative systems to guarantee continuity along their implementation. As result, the aim to achieve sustainability by involving all relevant stakeholders (also from the civil society) remained mainly unimplemented. Still concerning sustainability, as pointed out above, there is the possibility that one or more of these strategies may have been interrupted or not been implemented at all.

When geographical and cultural differences (C4) were considered (as in the case of the Spanish and Slovenian strategies), this was done in terms of distinction between urban and isolated/rural areas (rather than between regions), with the aim of strengthening regional economies, services accessibility, and co-housing initiatives. In the Irish strategy the activity patterns for older people are promoted in consideration of the cultural, economic and social life of the local communities.

Providing opportunities for AA by taking into account inequalities experienced by older people (C5) was considered in almost all strategies, mainly through economic, social protection (in Latvia and Malta) and health (in Slovenian) perspectives. In some cases (Ireland, Bulgaria and Spain), these dimensions were considered through a more comprehensive and cross-sectoral approach in terms of equal opportunities for dignified and adequate ageing.

The intergenerational and life-course perspective issue (C6) was included in almost all strategies, with the exception of the Latvian and the Bulgarian ones. However, especially in the Irish strategy, intergenerational cooperation seems to be more concretely considered in a life course perspective, forming positive attitudes towards AA from early ages through the family and the educational system.

Except for the Irish and the Czech ones, none of the considered strategies tried to avoid a too productive-oriented approach (C7). This is especially true for Latvia, where the strongly employment-oriented approach of the strategy and the related rapid growth of employment levels among older people are more likely to be explained by an attempt to overcome poverty rather than by progresses in AA overall [67]. Instead, in the Irish national strategy, employment is considered only as an opportunity among many others (e.g., civic engagement, cultural, spiritual, leisure and learning opportunities) for the continued involvement of people as they age in all aspects of cultural, economic and social life according to their needs, preferences and capacities. Along the same line, in the Czech’s national strategy, employment is considered as a dimension among many others (e.g., social protection, civic engagement, healthy lifestyle, education) which can lead to the strengthening of intergenerational relations and the degree of integration and participation, in order to generate an enabling social environment for seniors.

## 4. Aim of the Study

The existing national experiences inform about the difficulty to move from theory (the concept) to practice (its operationalization through policy making) regarding active ageing [15]. This is due to a lack of the employment of a model to operationalize the AA concept, by trying to deal with all the main challenges identified that the operationalization involves. In this study, the original model to comprehensively address the main challenges suggested as shown in Figure 1, is applied to provide recommendations for policy making in this field.

This model and its application are urgently needed, since the experiences of national active ageing strategies (or similar initiatives) described above, indicate that although some of the identified challenges were broadly addressed by the national strategies under study, their full and organic coverage was not present in any of them, suggesting a need for improvement in this respect.

In light of this, the purpose of this study is, for the first time, to operationalize and implement a comprehensive conceptual framework that considers all the main challenges identified in the literature for producing recommendations for policy making in the field of active ageing at the national level. This model should provide an example of good practice, to be applied in other context in the future. In generating the recommendations, a further aim is to stimulate commitment among the stakeholders by favoring the convergence of various points of views towards a shared perspective, facilitating the common action and the subsequent shift of paradigm [68]. This is in also line with Aspalter and Walker’s view (2015) [69], who urgently called for an adoption of a practical stance on how to design, pursue, measure and evaluate contemporary social policies on AA that take in consideration lacunae brought by the unpreparedness, ill-preparedness, not-yet-preparedness, and not-yet-fully-preparedness of governments, political parties, and civil society in meeting the needs and requirements of ageing populations.

## 5. The Ageing Situation in Italy

The model is applied in Italy, where over the last 50 years the ageing of the population has been one of the fastest among the most developed countries. In fact, with 60.8 million inhabitants, Italy has the largest proportion of older people (aged ≥ 65) in Europe (23.2% in 2020), and it is estimated that in 2050 the proportion will amount to 35.9% of the total population, with an average life expectancy of 82.5 years (79.5 for men and 85.6 for women) [70].

Population ageing in Italy is due to a number of reasons, mainly being represented by baby boomers growing old, increase in longevity, and low birth rate. See: ISTAT. Il futuro demografico del Paese. Previsioni regionali della popolazione residente al 2065. ISTAT 2011. Available online: http://www.istat.it/it/archivio/48875 (accessed on 30 November 2021). Furthermore, although international migration has increased in recent years, the addition of the foreign segment of the population has neither compensated for, nor significantly curtailed, the population ageing phenomenon [71]. Population ageing has a substantial impact in terms of economic sustainability, for example, the social protection system, due to a progressive shrinking of the workforce, and high incidence of pension spending in the overall resources allocated to welfare (despite recent reforms of the pension system adopted measures to tackle this trend, mainly by increasing retirement). There is also the (growing) issue of Long-Term Care of older people, in a context, on the one hand, of a welfare state based on a family-based welfare model with very limited public responsibilities [72]; on the other hand, of transformation of family structures, where the older generations continue to be the main pillar of the informal welfare system, both by supporting the income of families, and in caring for grandchildren or other children (+4.3% compared to 2010) See: INAPP. Report for Italy for the implementation of the MIPAA/RIS strategy 2018/2022. INAPP 2021 [73].

The evidence above clearly indicates the urgency of comprehensively promoting policy making in the field of active ageing at the national level in Italy, which was not recognized as a priority until 2019 [74].

## 6. Operationalization of the Model in Italy

At the beginning of 2019, as the first initiative undertaken in Italy at the national policy level for addressing the ageing challenge and the potential of AA, a 3-year Plan of Action (PoA) project was designed in compliance with the model of policy cycle [75] as pilot experience in view of a possible launch of a national AA strategy. The initiative was based on a 2018 agreement between the Department for Family Policies at the Presidency of the Council of Ministers of the Italian government, and the National Institute on Health and Science on Ageing (IRCCS INRCA), a public national organization funded by the Ministry of Health having the mission to conduct interdisciplinary gerontological and geriatrics research on ageing. The PoA was also supported by both the Ministry of Labour and Social Policies and the National Institute for Public Policy Analysis (INAPP), in their role of UNECE National Focal Points on ageing in Italy [76].

One of the main aims of the PoA was to produce research-driven recommendations for policy making in the field of AA, taking in consideration the challenges mentioned above.

### 6.1. Building the Coordination Tool

Methodologically, the first step was to build the stakeholder network by both involving representatives of public administrations (i.e., policy makers, managers and officers) and representatives of the relevant civil society organisations (associations and federations of older people, pensioners’ unions, academia, and other stakeholders). According to challenges 2 and 3, this was deemed as necessarily the first step to be taken, to allow that recommendations be built together from the beginning in a shared and co-decisional pathway through both in-person and on-line meetings, and through continuous interaction and feedback via e-mail. Given the scarcity of co-decisional tools regarding policies for older people in Europe [20], and the great importance of involving multiple actors and users assumes, in order to address this challenge, the stakeholder network was intended to be concretely engaged in all stages of the initiative, to take into account their different opinions, motivations, needs and concerns throughout the whole process [8]. This, on the one hand, avoided the risk of a top-down imposition of the AA concept by stimulating also the civil society to take action from the bottom-up. On the other hand, it pursued sustainability of collaborative work for policy making in this field. The stakeholder network involved representatives of around 100 Italian organisations, overall.

In order to consider the aspect of cultural diversity (C4), the stakeholder network included, in a multi-level perspective, representatives at both the national level and at the regional level. The importance of investigating regional differences in terms of AA has been underlined, among others, by Breza and colleagues (2014) [77], and by Marsillas Rascado (2019) [78]. As has been noted, social and cultural settings contextualise individual ageing, and vary considerably in terms of the demands they involve and the opportunities and resources they offer [79]. Italy is a country with great regional variation. Regional differences are pronounced since they are rooted in historical territorial divisions and autonomies [80], which result in different economic, social, cultural and political contexts, making evident the backwardness of the Southern part of Italy [81]. According to a recent study [34], five different groups of Italian regions have been found to demonstrate regional differences in the field of active ageing. Taking this into consideration, the idea was to produce recommendations for policy making general enough to allow their use both at the national and at the regional level, each level being capable to apply them starting from a different state-of-the-art, through renewable short-term objectives, in order to improve policy making according to the specific situations, competences and contexts. Accordingly, and in line with Edelenbos (1999) [82], the stakeholder network included representatives of all the relevant national government bodies (i.e., ministries and other relevant national public institutions), and of all the 19 Italian Regions and the two Autonomous Provinces, as well as representatives of major national civil society organizations, which were in contact and worked together to their regional branches. This would guaranteed a multilevel coordination of the actions, by strengthening both political and scientific commitment. More specifically, the participated approach provided a well-structured network that constantly exploits and transfers good practices [76], allowing the collection of sufficient data and information for translating the available knowledge into new policy, by making recommendations [83].

In order to guarantee a non-sectorial approach and the systematic participation of a wide range of policy areas, in line with the mainstreaming ageing perspective (C2), the institutional side of the stakeholder network included, at the national level, representatives of most of the governmental ministries and, at the regional level, representatives of most (regional) governmental departments.

As for the international framework (C1), in line with the conceptual framework previously discussed, the PoA was largely based on the MIPAA by the UNECE. The MIPAA was set in 2002 and consists of a global political framework for responding to the opportunities and challenges of population ageing aimed at improving the quality of life of older persons [84], with its review and appraisal taking every five years (i.e., RIS). Even if it is not a binding tool, it provides opportunities to Member States to reconsider national policies on ageing according to a life course approach, aimed at social inclusion of all generations [84].

Within the activities of the UNECE Standing Working Group on Ageing (SWGA), in 2019 nine out of the 17 Social Development Goals (SDGs) set within the 2030 Agenda for Sustainable Development [9], were added at the side of the 10 commitments set by the MIPAA [48]. The latter is a tool which stands alongside and supports the WHO framework on AA, the organization having also underlined the clear connection between SDGs and population ageing [85]. This framework was deemed as more comprehensive and organic than the AAI framework (since it also encompasses it). While the latter may be a very useful tool, among others, to inform policy making in this field, it may provide a partial view of the phenomenon when employed alone [34,65,66].

In line with the MIPAA commitment 2, which was intended to ensure the full integration and participation of older people in society by providing wide AA opportunities among which older people could freely choose based on their preferences, motivations and predispositions, a general and flexible definition of the activities that may be encompassed in this concept was adopted. Work, social, learning, leisure activities of older people, included those in the following spheres: social participation, training and lifelong learning, paid work, culture and tourism, sports and leisure, informal care (caregiving and grandparenting), agriculture and gardening, civic commitment and volunteering, co-housing, as well as any other area concerning the activation of older people, in order to guarantee the exploration of a large range of them.

**Figure 2:**
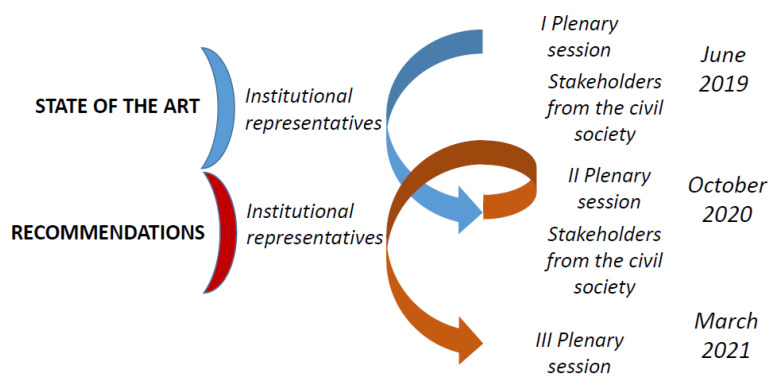
Pathway to evidence-based recommendations for policy making.

Figure 2, describes the steps towards the recommendations, moved after building the stakeholder network as described above.

### 6.2. Working on the Framework

The second methodological step was moved in June 2019 when the representatives of the organisations mentioned above, composing the stakeholder network, met for the first time (in person, in Rome). On this occasion was agreed, among other issues, the general structure of the project and the role of the stakeholder network within it. One of the points discussed during the first plenary session, and followed-up later through feedback via e-mail, was the framework represented by the ten MIPAA commitments and the nine SDGs composing it, and in particular the systematisation of them to manage duplications and to group similarities. At the end of the process, the framework assumed a 12-section structure (Table 5) where all the 19 MIPAA commitments and SDGs concerned were represented and related with the project perspective, that is, the perspective of AA.

Once the framework was agreed, the stakeholder network, together with the research team, agreed on the work plan and the interconnected actions, aimed to produce, in a joint way, the recommendations. This involved first to investigate the state of the art and, as the second step, to produce recommendation based on the analysis of the state of the art (Figure 2).

### 6.3. Studying the State of the Art of Public Policies in the Field of Active Ageing

Between 2019 and 2020, the research team conducted a systematic review of public policies on AA at national and regional level. Researchers carried out a primary and secondary data collection for each national and regional institution, including document review (e.g., laws, policies, statistics, reports) and (group and individual) interviews with managers and officers in charge of supervising AA policies. Fifty-four case study reports were produced [86]—one per each institution—by using standard template and reporting methodology. Cross-cutting analyses of all case study reports were carried out to provide an overview of the state-of-the-art on AA in Italy [87] as well as to monitor the alignment of AA policies with international policy frameworks, i.e., the MIPAA/RIS, the European Pillar of Social Rights and the SDGs [74].

As the data collection occurred until February 2020, the state-of-the-art analyses provided a picture of AA policies before the COVID-19 emergency and crisis response.

Both individual and cross-cutting reports were validated at different points in time with the policy officers and the stakeholders network (including also representatives from civil society and academia). The main results of the analysis of the state of the art were discussed in the second (online) plenary session, and later reported by Barbabella and colleagues [74,87].

### 6.4. Towards an Improvement of the State of the Art: Realising Recommendations for Policy Making

After the investigation of the state of the art, in compliance with the second step (“design and recommend”) of the policy analysis framework made by Mayer et al. (2004) [83], policy recommendations (available in Appendix A) were developed.

A policy recommendation is a written policy advice that serves to inform people (e.g., politicians, public officers) who are faced with policy choices, on particular issues. This is considered a valuable tool, based on strong evidence, to solve a public policy problem taking into account the existing best practices [88]. To ensure the efficacy of the recommendations, it is necessary to consider specific limitations, in terms of sustainability, imposed by the socio-economic context in which recommendations are released [88]. In line with the concept of social innovation [26], in producing recommendations, it is of outmost importance to cooperate among all the relevant stakeholders (i.e., policy makers and representatives from the civil society).

Following Majeski and Sylvan (1999) [63], in this study, policy recommendations are considered as main lines which serve to reopen the debate on a specific subject and thereby initiate a policy making phase. They are produced by comparing the effects of different policy alternatives [83], in line with the principles of the Evidence-Based Policy Making (EBPM) [89].

This process was methodologically developed through two main stages:

1. The project team analysed the state of art of AA policies [74,76], drown up through a participated approach with the stakeholder network of the PoA, and based on one national report and 35 individuals (i.e., organization-based reports concerning each of the Ministries, Departments of the Italian Presidency of the Council of Ministers, Regions and Autonomous Provinces, i.e., public administrations—involved in the project).
2. Organizations represented in the stakeholder network provided, individually, written feedback, at different levels of the process (on draft documents of the recommendations) and, in particular, a first input was delivered by the network in November-December 2020 through a web-consultation in the form of a self-administered questionnaire. The questionnaire is available for consultation here: https://forms.gle/rxFidDkQXnSsejab9 (accessed on 12 October 2021). The questionnaire was made of open questions designed to integrate the national report of the state of art with potential undervalued aspects.

Additional feedback on the first draft of the recommendations (February 2021) and later, during the third online plenary session in March 2021, with general observations, integrations and proposals for amendment, was provided. The research team took into account the critical and interpretative contributions received and then provided a final version of the recommendations [90].

Importantly, when the project started, and during the fieldwork to investigate the state of the art of national and regional public policies in the field of AA, the COVID-19 pandemic had not broken out. At the end of 2020, when it became manifest, the project could not ignore its impact in an AA perspective. For this reason, consistent with the UNECE policy brief n. 25 issued in November 2020 [91], a further (thirteenth) topic was added to the initial project framework, namely the “management of older people in emergency situations”, with the contingency being represented by the pandemic. This implied to update the state of the art concerning this particular topic, through a dedicated set of questions included in the questionnaire mentioned above (i.e., the web-consultation).

In general, the 28 policy recommendations were obtained (for a consultation of the recommendations and of the short-terms goals identified up to now, see the Appendix A) at the end of the co-decisional process, in the 13 AA dimensions mentioned above, and were formulated in order to be general enough for, on the one hand, possible application of them in every geographical and political Italian context/level, independent of the state of the art concerned. On the other hand, for being valid in time, the enunciation of too direct aims would have caused obsolescence of the recommendations once aims were achieved. Direct aims related to each of the recommendations should have been established as short-term goals to be continuously renewed, once achieved.

## 7. Conclusions

In this article we provide and apply a new model for producing recommendations for policy making and policy implementation in the field of AA. The main challenges to be considered for a proper operationalization of the AA concept are identified. The exploration of the operationalization process of the AA concept in different European national strategies provided in Section 3, demonstrates that none of them comprehensively addressed the main challenges identified, thus demonstrating the presence of flaws in the applied models. To fill this gap, through this study, a comprehensive model was elaborated and, for the first time, applied at the Italian national policy level for producing recommendations for policy making in the field of AA, which constitutes a pilot experience in view of a possible national AA strategy.

This model intended to consider and include all the relevant points, in order to guarantee a proper operationalization of the AA concept, to favor sustainability of AA policy making by ensuring a mainstreaming ageing approach at the public policy level and a stakeholder wide participation through co-decisional tools [92]. This allows adoption of a multi-level (national-regional-local) perspective, which considers cultural and geographical diversity in order to strengthen the connection between governments and territories [93]. The adoption of the framework represented by the MIPAA and the SDGs, allowed inclusion, among others, of meaningful domains of activation of older people rather than just employment [94]; a life-course perspective focusing on intergenerational relationship [95], and consideration of inequalities (due to the exposure to multiple health, environmental and social risks or barriers) in terms of possession of various kind of resources [96,97].

The main interest of this study was to focus on the development of a model for producing recommendations for policy making and policy implementation in the field of AA, rather than on the specific contents of the recommendations produced. Theoretically speaking, this is due to the fact that whether the model is recognized as properly operationalizing the concept of AA, the contents of the recommendations should not be one-size-fits all. Rather, they (and the related short-terms aims) should be contextualized and specific, depending on the state of the art on the matter, and the point of view, in co-decisional terms, of all the relevant stakeholders (from the government and the civil society at the various levels: national, regional, local). While the general model could be replicated in different national (and regional) contexts, it is less likely that the specific content of the recommendations (and short-term goals) produced in Italy could also be applied to other contexts as the needs, infrastructure, culture and perspectives of AA change from country to country [92].

As for the Italian context, future studies should concentrate more specifically on the contents of the recommendations issued for Italy, and on the possibility of giving them concrete implementation by evaluating the impact they have on policy making. Relative to the main short-term objectives set to implement the recommendations are the identified ambitious aims to be reached; for example the establishment of a “National Observatory on Active Ageing” (under R1) that should guarantee the sustainability of the coordination tool set specifically for this pilot experience. Other short-term aims are the production and enforcement of a national law promoting active ageing (under R3) and (see R4) being sure that the existing Regional Laws on this matter are implemented instead of remaining unimplemented (as it is the case in some regional contexts).

Recommendations 24 to 26 concern the topic of the management of older people in emergency situation, the contingency of this being represented by the COVID-19 pandemic. Seeing this topic in an active ageing perspective, the recommendations wanted to underline not only the needs of older people but also their proactive role to respond to the emergency (see R24). Certainly, the pandemic affected, in some way, schedules related to active ageing. Just sticking to the experience described in this paper, there has been a time dilation (although it has been successfully managed) after the phase of the state-of-the-art analysis, and most of the activities previously planned to be in-person, were carried out through video-conference/s due to the pandemic. More broadly, all the policy areas of AA considered through the recommendations, seem to have suffered the negative impact of the pandemic, and in particular the areas of health and well-being, mainstreaming ageing, social protection, and participation of older people in society [90]. Therefore, especially these areas should be addressed by political intervention in the short-term. Even the future impact of the pandemic on the policies related to AA, are currently difficult to fully grasp. It is clear that policy intervention should also concern long-term aims, since demographic effects of the pandemic are expected. Indeed, due to the pandemic, on one hand, Italy has experienced a reduction in fertility, and on the other hand, a reduction in life expectancy [98]. What has become clear in the last two years is that people over the age of 65 have been those most vulnerable, considering that the pandemic has heavily overloaded health services, favoring situations of inadequate treatment and violation of the rights and dignity of seniors [87].

As a limitation this study, the recommendations produced have not been recognized as a binding tool through governmental documents. So, even if they are may be used, among others, by policy makers at various governmental levels, they will be applied on a voluntary basis. Thus, the operationalization of the policy recommendations depends on the strong commitment of all relevant actors. In addition, the recommendations are thought to be general enough to allow their use both at the national and regional level, each level being capable to apply them to improve policy making according to the specific situations, competences and contexts. In light of this, it is of paramount importance to contextualize the recommendations, and to set short-term objectives that must be constantly renewed once they are achieved. Therefore, at the governmental level, a necessary step for the adoption of an active ageing national strategy involves making the recommendations mandatory. Another challenge is to guarantee the sustainability of the initiative. To do this, it is important to keep active ageing high on the national political agenda. In this respect, the expectations are promising, in which the vast, structured and highly motivated network of stakeholders, established through this experience, will ensure continued commitment, also in view of the electoral power of the older population (which in 2020 represented 23.2% of the Italian population and, therefore, an important segment of voters).

Next research steps will require the study of possible replication of the present model in other national contexts. As already underlined above, concerning the Italian experience described in this study, a further research step would be to analyse the impact and effectiveness of the model, i.e., the extent to what the recommendations may affect policy making at both the national and at the regional level.

## Figures and Tables

**Table 1:** Challenges and possible solutions regarding policy making in the field of active ageing.

	Challenge	Possible Solution
C1	Narrow policy approach	To adopt a comprehensive international policy framework
C2	Policy silos	To mainstream active ageing in all policy fields
C3	Top-down approach	To involve relevant stakeholders through co-decisional tools
C4	One-fits-all approach	To respect cultural diversity by differentiating policy solutions according to the contexts
C5	Limited equal opportunities for older people	To provide opportunities for all, considering inequalities
C6	Age-segregation approach	To adopt a life-course perspective through promoting intergenerational relationships and solidarity
C7	Mainly work-oriented approach	To promote participation in all social domains

**Table 2:** The international policy framework.

MIPAA Commitment	SDGs Linked to the MIPAA
1 Mainstreaming ageing	1 No poverty
2 Integration and participation	3 Good health and well-being
3 Economic growth	4 Quality education
4 Social security	5 Gender equality
5 Labour markets	8 Decent work and economic growth
6 Lifelong learning	10 Reduced inequalities
7 Quality of life, independent living and health	11 Sustainable cities and communities
8 Gender equality	16 Peace, justice and strong institutions
9 Support to families providing care and intergenerational solidarity	17 Partnerships for the goals
10 Regional co-operation	

Data source: UNECE [23]—https://unece.org/DAM/pau/RIS.pdf (accessed on 7 December 2021); United Nations [11]—https://www.un.org/ga/search/view_doc.asp?symbol=A/RES/70/1&Lang=E (accessed on 7 December 2021).

**Table 3:** Overview of national AA strategies in Europe.

Country	Title	Year/s	Recommendations ^1^
Bulgaria	National Strategy for Active Ageing in Bulgaria [49]	2019–2030	No
Czech Republic	National Action Plan Supporting Positive Ageing [50]	2013–2017	No
Ireland	The National Positive Ageing Strategy [51]	2013	No
Latvia	Developing a Comprehensive Active Ageing Strategy for Longer and Better Working Lives [52]	2014–2016	Yes
Malta	National Strategic Policy for Active Ageing [53]	2014–2020	Yes
Slovenia	Active Ageing Strategy [54]	2017	Yes *
Spain	Framework of action for older persons [55]	2014	Yes **

^1^ Yes = developed, No = not developed; * Guidelines; ** Proposals.

**Table 4:** Adherence of European AA strategies to the conceptual framework.

Country	C1	C2	C3	C4	C5	C6	C7
Bulgaria	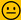	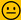	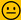	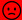	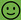	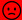	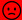
Czech Republic	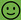	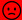	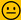	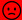	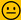	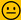	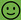
Ireland	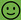	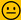	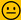	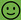	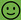	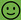	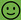
Latvia	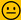	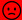	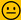	N.A.	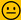	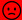	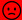
Malta	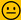	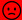	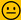	N.A.	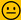	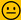	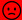
Slovenia	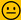	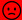	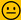	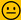	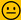	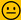	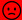
Spain	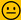	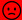	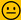	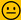	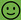	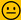	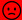

Legend: 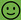: Covered 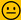: Partially covered 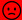: Uncovered.

**Table 5:** Subjects and purposes of the national PoA on active ageing.

Subject	MIPAA	SDG	Purpose
1 Mainstreaming ageing in all policy fields	1		Overcoming the sectoral visions and fostering a system perspective in order to address the challenges related to ageing. In the field of active ageing, positive experiences both at the national and local levels, are those that promote and put into practice an inter-ministerial or inter-departmental (at the regional level) collaboration, overcoming the classic approach that delegates the production and management of interventions in this area to social and health policies.
2 To ensure the full integration and participation of older people in society	2		To promote the integration and participation of older people in society, in all areas of active ageing, without exception, to ensure that all possible opportunities are provided, among which older people can freely choose on the basis of their preferences, motivations and predispositions.
3 To strengthen the partnership		17	To involve relevant stakeholders in all processes (from the production of policies on active ageing, to the implementation of services and related monitoring) with consultation and co-decisional tools. The subject is strongly linked to the previous two, as it strengthens the integration and participation of older people in society by integrating consultation and co-decision into mainstream ageing tools.
4 To promote the fight against inequalities and poverty, fostering a fair and sustainable economic growth	3	1;10	Inequalities are considered as barriers that prevent access to active ageing paths, which must be guaranteed to the entire older population regardless of differences in cultural resources, income, education and health, precisely in order to reduce them. This vision, therefore, does not include the strictly welfare part of older people in need of social and health care, but rather those cases in which inequalities are given by differences in access to resources and the ability to achieve their own life goals, with respect, for example, to specific socio-economic conditions.
5 Modification of social protection systems in response to demographic changes and their socio-economic consequences	4		While generally this commitment is exclusively traced back to the issue of pensions, in reference to active ageing, by social protection it is meant something broader, which, in addition to the theme of combating inequalities and poverty (see the previous point), it includes the construction and redefinition of a new welfare system.
6 Adapting the labor market to respond to the economic and social consequences of an ageing population	5	8	Employment is considered as an important dimension, among those that pertain to the concept of active ageing promoted through commitment 2. Ensuring participation of older people in this area is a necessity for both institutions and companies, in particular in managing the effects of the extension of working life both on the production process and as a function of the mechanisms of intergenerational exchange and transmission of knowledge.
7 Promotion of lifelong learning and adaptation of the educational system in response to economic, social and demographic changes	6	4	Poor levels of education can have negative repercussions throughout the life span, becoming an obstacle to the pursuit of healthy and active ageing. The theme of education and learning also has a considerable impact on other important dimensions. For example, that of income (as low education is generally correlated with low income), with direct repercussions on the type of work performed, on the state of health and on the quality of life.
8 To promote initiatives to ensure quality of life, independence, health and well-being.	7	3	Health and quality of life are key elements in the field of active ageing, which, as a result, contribute to obtaining positive feedback in this sense, so that benefits in terms of health and quality of life are also enjoyed by people with a health deficit. On the other hand, greater health problems imply greater problems in accessing active ageing.
9 Enhancement of the gender approach in a society characterized by demographic ageing	8	5	The issue of the gender approach, highly regarded by MIPAA and the 2030 Agenda for sustainable development, can be considered as a specific declination of the more general problem of inequalities.
10 To support families providing care to older people and to promote intergenerational solidarity	9	16	Support provided to families, in relation to care activities, should be not only a responsibility of the governmental bodies that provides these services, but also of the community in general, with a view to solidarity. To consider the life cycle perspective is critical for several reasons. Therefore, it is also necessary to think about active ageing to prepare future generations to face old age in the best possible way.
11 Sustainable cities		11	In order to guarantee to older people access to all the opportunities for active ageing, it is important to consider the methods of access to the services and active ageing paths which are present in the area, in terms of organization of transports, adequacy of housing and infrastructure.
12 Cooperation for the promotion and full realization of the Regional Strategy for the implementation of the MIPAA	10		The strategy for the implementation of the MIPAA (Regional Implementation Strategy—RIS) consists in making sure that everything that has been discussed through the previous points is concretely realized.

## Appendix A. Recommendations for the Adoption of Active Ageing Policies in Italy

**Table A1:** MIPAA Commitment 1: Mainstreaming ageing.

**Recommendation n.1**
To provide long-term tools for coordination, analysis, planning and monitoring of active ageing policies at national level, by involving all the Ministries, Departments at the Presidency of the Council of Ministers, Regions and Autonomous Provinces.
**Recommendation n.2**
To provide long-term tools for coordination, analysis, planning, implementation and monitoring of active ageing policies at regional level, by involving all regional departments/services, as well as other important institutional regional actors.
**Short-Term Objectives:**
(a) Creation of a National Observatory on Active Ageing. (b) Creation of regional tools such as “Permanent working tables on active ageing”, or similar.

**Table A2:** MIPAA Commitment 2: Integration and participation.

**Recommendation n.3**
To ensure full integration and participation of older people in society at the national and regional levels through specific and adequate laws and regulations
**Recommendation n.4**
To ensure actual (rather than just remaining on paper) full integration and participation of older people in society as provided by laws, decrees, resolutions and other regulatory documents.
**Short-Term Objectives:**
(a) Approval an implementation of a national framework law, for a comprehensive promotion of active ageing. (b) Approval an implementation of regional laws or similar regulations, for a comprehensive promotion of active ageing.

**Table A3:** SDG 17: Partnerships for the goals.

**Recommendation n.5**
To ensure that, beyond representatives of institutional/governmental bodies, both at the national and the regional level, also all relevant stakeholders (from the third sector and civil society, the academic-scientific sector, etc.) are included in long-term tools for the analysis, planning, implementation and monitoring of policies in the field of active ageing, in order to guarantee co-decisional participatory mechanisms.
**Short-Term Objectives:**
(a) To guarantee sustainability over time of the stakeholder network created at national level within the project “National multi-level co-managed coordination of active ageing policies”. (b) To create or activate (in case they exist but still they remain on paper) a stakeholder networks in each Region/Autonomous Province.

**Table A4:** MIPAA Commitment 3: Reduced Inequalities; MIPAA Commitment 3: Economic growth; SDG 1: No poverty.

**Recommendation n.6**
To promote policies to combat inequalities and poverty, in order to guarantee the possibility of ageing actively also to older people with few resources available in terms of health and socio-economic conditions. Opportunities should be provided not only in terms of economic help, but also in terms of activation in the various domains of active ageing, according to the characteristics of the territory and promoting the development of digital skills among older people.
**Short-Term Objectives:**
(a) To strengthen, in the area of welfare services of the Local Authority, the implementation of counters for, taking into account inequalities between them, accompanying older people towards active ageing paths. (b) To promote the development and coordination of national and regional initiatives, aimed at reducing the digital divide of the older population, and at promoting digital literacy, since the latter are actions capable of combating inequalities and fostering active ageing; and of guaranteeing independence, empowerment and equity of access to services and information, in response to individual needs (digital citizenship).

**Table A5:** MIPAA Commitment 4: Social security.

**Recommendation n.7**
In order to promote adequate social protection in response to demographic changes, and their socio-economic consequences, it is necessary build a new welfare system through the development of a multi-level institutional governance, both at national and regional level, which integrates the perspective of ageing throughout life, and in the different life spheres.
**Short-Term Objective:**
(a) Realisation of a system of proximity services for protection and social integration of older people who live in disadvantaged areas, for example: mountain villages, inland areas and suburbs.

**Table A6:** MIPAA Commitment 5: Labour markets; SDG 8: Decent work.

**Recommendation n.8**
To promote, at all levels and alongside possible existing ones, the implementation of policies stimulating age management initiatives both in the private and the public sectors. These initiatives are necessary to guarantee:
To mature workers: the development of more opportunities and quality of the working conditions, resources and skills, including forms of work regulation and organization, to enhance intergenerational differences. To employers: the achievement of better economic results, also in terms of corporate social responsibility, by at the same time providing older workers with a better work climate, thus improving their work motivation, satisfaction and productivity, enhancing the potential of intergenerational teamwork.
**Recommendation n.9**
To promote active labor market policies at national and local level, which should be functional to vocational retraining, to skill-updating and to work reintegration of all those who wish so (mature unemployed and/or disadvantaged individuals; retired older people, etc.).

**Table A7:** MIPAA Commitment 6: Lifelong learning; SDG 4: Quality education.

**Recommendation n.10**
To strengthen lifelong learning within a global strategy with the “Plan for the development of skills of the adult population” as a strategic tool, to represent a solid reference base for guiding targeted interventions that could be also funded within the European programming.
**Recommendation n.11**
To strengthen lifelong learning by promoting intergenerational knowledge exchange in a bidirectional way across various domains (e.g., areas, for example, passing on of knowledge by older people; passing on of digital skills by younger people).

**Table A8:** MIPAA Commitment 7: Quality of life, independent living and health; SDG 3: Good health and wellbeing.

**Recommendation n.12**
In order to improve the implementation of preventative tools, to provide training programs and policies able to strengthen competences in the community, and also including the promotion of active ageing among other tools.
**Recommendation n.13**
To create bridges between the health (doctors, geriatricians, health workers in general) and the gerontological (gerontologists, professions relating to the social aspects of ageing) perspectives, also through a two-way training for the operators of these two fields, in order to exploit and coordinate in a more effective way the activities developed in the area of active ageing.

**Table A9:** MIPAA Commitment 8 and SDG 5: Gender equality.

**Recommendation n.14**
To consider the issue of gender inequalities, in all areas of active ageing.
**Recommendation n.15**
To plan tools to implement gender-related initiatives required by regulations.
**Recommendation n.16**
To promote specific policies and initiatives to combat violence, abuse and discrimination against older women, also in light of the social transformations of the family under way, thus fostering their activation in the various active ageing domains.

**Table A10:** MIPAA Commitment 9: Support to families providing care; SDG 16: Peace and (intergenerational) justice.

**Recommendation n.17**
To facilitate caregivers in the access to all relevant information they need (including information on how to carry out care activities in relation to the specific diseases suffered by older people), through the creation of specific digital platforms (or the development of those already existing), for also providing training and information on the management of the disease.
**Recommendation n.18**
To promote the recognition of the rights and of the activities carried out by the caregiver, in the perspective of combating inequalities, including those related to health; promoting a gender approach, also creating a network in the community in order to facilitate the relationships between families and public and private services, also considering elements of training for family carers.
**Recommendation n.19**
Through services and devices, to provide older people and their caregivers the possibility of combining the illness and the care activity with their life-project within the community, e.g., relative to the work for the labour market or to other active ageing domains (learning, leisure and cultural activities, volunteering, etc.).
**Recommendation n.20**
It is necessary to encourage intergenerational dialogue in a positive and bidirectional way, also to the aim of promoting the life-course perspective.
**Short-Term Objective:**
(a) Creation of a register of non-self-sufficient older people

**Table A11:** SDG 11: Sustainable cities and communities.

**Recommendation n.21**
To promote initiatives to facilitate mobility and access to community services (including educational ones) of older people, both in terms of time-flexibility and of adaptation of public transportation, as well as of pedestrian and cycle walkways.
**Recommendation n.22**
To promote both the development of enabling technologies and the adaptation of building and urban planning standards, for the reorganization of living spaces, even in co-housing situations, in an active ageing perspective. To also adopt criteria for the assessment of the quality of the houses of older and frail people.
**Recommendation n.23**
To promote the different types of co-housing (e.g., inter and intra-generational, neighborhood co-housing, eco-rural villages, social housing, etc.) in older age and innovative strategies of urban regeneration, in order to promote active participation in social life.

**Table A12:** Older people in emergency situations.

**Recommendation n.24**
To provide plans taking into account both older people’s needs and the contribution that they can offer in all the stages: preparation, support and response to the emergency.
**Recommendation n.25**
To promote data collection and data process related to health and living conditions of older people during emergency situations, to encourage the implementation and the transferability of good practices.
**Recommendation n.26**
To consider the condition of older people in emergency situations, in a cross-cutting way with respect to the MIPAA commitments and the Sustainable Development Goals previously discussed.

**Table A13:** MIPAA Commitment 10: Regional co-operation.

**Recommendation n.27**
To keep active ageing at the top of the political agenda at the national, regional and local governmental level, also via media, through an effort by all the relevant stakeholders.
**Recommendation n.28**
To take into account, in all the relevant laws and policies at all levels, within public, private and third sector organisations, including by older people themselves, each according to the respective competences and available resources, all the recommendations expressed in this document, to guarantee that the rights of older people are respected.
**Short-Term Objective:**
(a) To strengthen the available statistics about living conditions of older people.
